# Lipid Accumulation and Injury in Primary Calf Hepatocytes Challenged With Different Long-Chain Fatty Acids

**DOI:** 10.3389/fvets.2020.547047

**Published:** 2020-10-15

**Authors:** Bingbing Zhang, Wei Yang, Shuang Wang, Runqi Liu, Juan J. Loor, Zhihao Dong, Yingying Zhao, Xinru Ma, Cheng Xia, Chuang Xu

**Affiliations:** ^1^College of Life Science and Technology, Heilongjiang Bayi Agricultural University, Daqing, China; ^2^College of Animal Science and Veterinary Medicine, Heilongjiang Bayi Agricultural University, Daqing, China; ^3^Mammalian NutriPhysioGenomics, Division of Nutritional Sciences, Department of Animal Sciences, University of Illinois, Urbana, IL, United States

**Keywords:** non-alcoholic fatty liver disease, fatty acids, lipid accumulation, endoplasmic reticulum stress, negative energy balance, primary calf hepatocytes

## Abstract

Fatty liver disease is one of the most common disorders afflicting dairy cows during the postpartum period, and is associated with increased blood non-esterified fatty acid (NEFA) uptake by the liver. Major long-chain fatty acids (LCFA) in NEFA are palmitic (PA), palmitoleic (POA), stearic (SA), oleic (OA), and linoleic (LA) acid. In order to investigate the characteristics of lipid accumulation and injury caused by these NEFA, primary calf hepatocytes were isolated and challenged for 12 h with 1.2 mmol/L PA, POA, SA, OA, LA, or a mixture of these LCFA (NEFA). Compared with POA, OA, and LA, culture with PA and SA led to greater abundance of CCAAT-enhancer binding protein, glucose-regulated protein 78 mRNA, and stearoyl-CoA desaturase 1 mRNA along with greater concentrations of H_2_O_2_, malondialdehyde and reactive oxygen species (ROS). Although culture with POA, OA, and LA led to lower very low density lipoprotein (VLDL) concentration in cell culture medium, POA and OA led to greater concentrations of triacylglycerol, protein abundance of sterol regulatory element-binding protein 1c, fatty acid synthase, acetyl coenzyme A carboxylase 1, ApoB100, and sortilin 1 (SORT1). Compared with individual fatty acids, culture with NEFA led to an intermediate degree of lipid accumulation and hepatocytes damage. Overall, the data suggest that saturated fatty acids cause more severe oxidative and ER stress. However, unsaturated fatty acids cause serious lipid accumulation. Furthermore, a fatty acid balanced nutrient regulation was suggested useful improve liver health of transition period dairy cows.

## Introduction

Negative energy balance (**NEB**) is a subhealthy state commonly experienced by transition period cows as a consequence of insufficient dry matter intake to sustain the high energy requirements for maintenance of body functions and milk production. With severe NEB, a large number of non-esterified fatty acid (**NEFA)** produced by fat mobilization, usually lead to the occurrence of fatty liver (1). Fatty liver is the most-important metabolic disease afflicting dairy cows early postpartum (2). It is usually characterized by increased plasma NEFA concentrations, accumulation of lipid droplets in hepatocytes (3), and hepatomegaly and inflammation (steatohepatitis) (4). In rats, evidence indicates that oxidative stress and endoplasmic reticulum stress may promote steatosis, reactive oxygen species (**ROS**) production and development of non-alcoholic steatohepatitis (**NASH**) (5). Steatosis is an early stage of fatty liver, which leads to lipid peroxidation, inflammation, hepatic stellate cell activation, and fibrosis through intracellular NEFA accumulation (6). The high concentration of plasma NEFA in transition period cows contains a variety of fatty acids, with palmitic (**PA**), palmitoleic (**POA**), stearic (**SA**), oleic (**OA**), and linoleic (**LA**) acid being the most abundant (7–9). Clearly, these represent major fatty acid classes, saturated (**SFA**, PA, SA), monounsaturated (**MUFA**, POA, OA), and polyunsaturated (**PUFA**, LA). Some data from human studies indicate alterations in plasma long-chain fatty acid profiles among the healthy, non-alcoholic fatty liver and NASH (10), but it is unclear if the major fatty acids in the NEFA fraction of dairy cows have equal potency to induce hepatocytes lipid accumulation and injury.

Endoplasmic reticulum (**ER**) stress is associated with fatty liver, NASH and other inflammation-related diseases. It is a response to abnormal protein synthesis, folding, modification, or transport (11, 12). In liver *in vivo* and *in vitro* study of cow, ER stress can lead to fat accumulation leading to activation of the unfolded protein reaction (**UPR**) and alterations in lipid metabolism (4). Increased expression of ER chaperones such as glucose-regulated protein (**GRP78**) that cope with accumulation of unfolded or incorrectly folded proteins, is a marker of ER stress (13, 14). C/EBP homologous protein (**CHOP**) is the main signal protein that connects ER stress and hepatocyte injury (15). Endoplasmic reticulum stress can induce hepatic steatosis by reducing lipid outflow or catabolism (16). Present *in vivo* study confirms the occurrence of ER stress in dairy cows with severe fatty liver, *in vitro*, high fatty acids induced calf hepatocytes ER stress promoted lipid accumulation (4).

Sterol regulatory element-binding protein-1c (**SREBP-1c**) is a transcription factor that can regulate hepatic fatty acid synthesis. SREBP-1c target genes include acetyl coenzyme A carboxylase 1 (**ACC1**) and fatty acid synthase (**FAS**), which regulate liver fat accumulation and participate in the progression of hepatic steatosis and hypertriglyceridemia (17, 18). Very low density lipoprotein (**VLDL**) is one of the major lipoproteins that transport endogenous triacylglycerol (**TG**) out of the liver (19). Apolipoprotein B100 (**ApoB100**) is the major protein in VLDL and is essential for loading of TG into the growing VLDL molecule (20, 21). Sortilin (**SORT1**) is a receptor for ApoB100, and regulates low density lipoprotein-cholesterol (**LDL-C**) levels by mediating lipid particle synthesis and transport (22). In contrast, proprotein convertase subtilisin/kexin type 9 (**PCSK9**) can reduce uptake and metabolism of LDL-C by promoting lysosomal digestion of LDLR (23).

In transition period cows, PA, POA, SA, OA, and LA are the most abundant fatty acid in plasma, some of which have demonstrated more potency in terms of altering lipid metabolism *in vitro*. For instance, OA induced more pronounced alterations in lipid metabolism in human hepatocytic cell lines than a mixture of NEFA underscoring potential differences in liver metabolism among long-chain fatty acids (24). Obviously, each FA can differentially affect the metabolism of liver. Our hypothesis was that long-chain FA would have different effects on lipid injury of cow hepatocytes dependent on the specific FA. Therefore, the primary aim of this *in vitro* study was to investigate alterations in lipid metabolism, oxidative stress and ER stress in calf hepatocytes incubated with various fatty acids.

## Materials and Methods

### Animal Care and Experimental Protocols

The study protocol was approved by the Ethics Committee for the Use and Care of Animals, Heilongjiang Bayi Agricultural University (Daqing, China). The animal studies were performed in accordance with the Guiding Principles of Animals adopted by the Chinese Association for Laboratory Animal Sciences.

The experimental animals were purchased from a farm in Heilongjiang Province (China). Newborn healthy female Holstein calves (*n* = 4, 1 d old, 40–50 kg, rectal temperature 38.7–39.7°C) were fasted for 24 h. After intramuscular injection of sodium thiopentobarbital (50 mg/kg) for anesthesia, they received an intravenous injection of 1,500 IU/kg heparin sodium for anticoagulation. The caudate lobes of the liver were obtained by surgical hepatectomy, and quickly moved to a super-clean bench. The animals were euthanized immediately by intra-venous injection of saturated potassium chloride (150 mg/kg of calf BW). Primary hepatocytes were isolated using a modified 3-step collagenase perfusion method as previously described (25). The perfusion solution A (140 m*M* NaCl, 6.7 m*M* KCl, 10 m*M* HEPES, 2.5 m*M* glucose, and 0.5 m*M* EDTA. pH 7.4; 37°C, 50 mL/min for 10–15 min) preheated at 37°C was used to rinse the blood surface of the caudate process surface, and the blood vessel of the cross section was leaked. The liver was then perfused with the same flow rate of 37° C pre-warmed perfusion solution B (140 m*M* NaCl, 6.7 m*M* KCl, 30 m*M* HEPES, 2.5 m*M* glucose, and 5 m*M* CaCl_2_, pH 7.4; 37°C, 50 mL/ min for 5 min)until the liquid became clear. Then, the liver was perfused with perfusion solution C (0.1 g of collagenase IV dissolved in 0.5 L of perfusion solution B, pH 7.2–7.4; 20 mL/min for 15 min) to dissociate liver tissue structure until the liquid became muddy. The digestion was terminated with 4°C pre-cooled fetal calf serum (**FBS**; Hyclone Laboratories, Logan, UT), the liver capsule was torn off with scissors and forceps, the tissue was cut, and the blood vessels and connective tissue were removed. The shredded liver tissue was resuspended in 4°C pre-cooled RPMI1640 medium and filtered sequentially with 100 mesh (150 μm) and 200 mesh (75 μm) cell sieves.

The suspension of hepatocytes was washed with basic medium (Hyclone Laboratories), centrifuged, and resuspended in the adherent medium, counted and seeded into a 6-well tissue culture plate (2 mL per well) at 1 × 10^6^ cells/mL using adherent medium (RPMI-1640 basic medium supplemented with 10% FBS, 1 μM of insulin, 1 μM of dexamethasone, 10 μg/mL of vitamin C) and incubated at 37°C in 5% CO_2_ for 4 h. Then growth medium (RPMI-1640 basic medium supplemented with 10% FBS) was changed every 24 h until the primary hepatocytes were cultured for 44 h.

In order to explore the different effects of single fatty acids and NEFA, we screened five fatty acids as the experimental group according to the composition of NEFA, which included OA, LA, PA, POA and SA (Sigma-Aldrich, St. Louis, MO). Stock NEFA solution was prepared by diluting individual fatty acids in 0.1 M KOH at 60°C. pH of the solution was adjusted to 7.4 with hydrochloric acid (1 M). The stock NEFA (52.7 mM) solution contained OA (22.9 mM), LA (2.6 mM), PA (16.8 mM), SA (7.6 mM), and POA (2.8 mM). The concentration of OA, LA, POA, PA and SA was 50 mM after dissolution with 0.1 *M* KOH, and stored at −20°C after filtration and sterilization. Fatty acids were diluted to 1.2 mM in RPMI-1640 basic medium and added to the 6-well tissue culture plate after 12 h of starvation for 12 h.

### RNA Extraction and PCR

Total liver RNA was extracted using TRIZOL (Invitrogen Corporation, Carlsbad, CA, USA). Total RNA was dissolved in diethylpyrocarbonate water. The RNA was transcribed into cDNA by an oligonucleotide primer using Reverse Transcriptase M-MLV (RNase H-). *GRP78, SREBP-1c, FAS, SORT1, ACC1, ApoB100*, and *SCD1* mRNA expression were assayed with an Applied Biosystems 7300 real-time PCR system using SYBR Premix Ex Taq I (F. Hoffmann-La Roche AG, Basel, Switzerland). Gene primers were designed using Applied Biosystems Primer Express software and are shown in Table 1.

**Table 1:** Sequences of primers used for real-time PCR amplification.

**Gene**	**Forward (5′-3′)**	**Reverse (5′-3′)**
β-actin	GCTAACAGTCCGCCTAGAAGCA	GTCATCACCATCGGCAATGAG
GRP78	GCATCGACCTGGGTACCACCTA	CCCTTCAGGAGTGAAAGCCACA
SREBP-1c	GCAGCCCATTCATCAGCCAGACC	CGACACCACCAGCATCAACCACG
FAS	ACAGCCTCTTCCTGTTTGACG	CTCTGCACGATCAGCTCGA
SORT1	TCCTTGGACCGACATCTCTACACC	TCGCATTCACTGTTCTCAGGCTTC
ACC1	TCCTGCTGCTATTGCTACTCCA	CAGTCCCCGCACTCACATAA
SCD1	CCGCCCTGAAATGAGAGATG	AGGGCTCCCAAGTGTAACAGAC

### Western Blot Assay

Hepatocytes were harvested and washed twice in ice-cold PBS. Total cellular protein was extracted using a protein extraction kit according to the manufacturer's instructions. Aliquots of 60 μg total protein were denatured for 5 min at 100°C and loaded on 10% sodium dodecyl sulfate polyacrylamide gels and electro-transferred onto polyvinylidene difluoride (**PVDF**) membranes. After incubation with blocking buffer, membranes were exposed to polyclonal GRP78 (78 kD, 1:250, sc-376768; Santa Cruz, CA), SREBP-1c (68 kD, 1:1000; NB100-2215; Novus, USA), FAS (273 kD, 1:1000; C2065; Cell Signaling, Danvers, MA, USA), SORT1 (85 kD,1:1000; ab16640, Abcam, Cambridge, MA), P65 (65 kD, 1:1000; D14E12; Cell Signaling; Danvers, MA, USA), ACC1 (265 kD, 1:1000; ab45174, Abcam, Cambridge, MA), CHOP (27 kD, 1:1000; L63F7; Cell Signaling, Danvers, MA, USA), PCSK9 (65–80 kD, 1:1000; 85813, Cell Signaling; Danvers, MA, USA), SCD1 (37 kD, Cell Signaling; Danvers, MA, USA). After washing and incubation with secondary antibody, immunoreactive bands were detected by enhanced chemiluminescence (Beyotime, China) solution. The imprinting was exposed with X-ray film to radiograph the spectral band, and the gray value of the spectral band was measured with bandscan software version 5.0 (prozyme Inc, San Leandro, CA). Protein abundance is reported relative to that of β-actin as a ratio of optical densities.

### Detection of Biochemical and Oxidative Stress Indices

Triacylglycerol content in cells was detected by TG test kit (Mnzyme method, Solarbio Life Sciences, Beijing, China), H_2_O_2_ and malonaldehyde (**MDA**) in cells were detected by H_2_O_2_ Assay Kit and Lipid Peroxidation MDA Assay Kit (Beyotime Biotechnology, Shanghai, China).

### Detection of Very Low Density Lipoprotein (VLDL) Content

The content of VLDL in the cells was detected by the Cattle Low Density Lipoprotein Assay Kit (Lengton Bioscience Co, Shanghai, China). Refer to the instructions of the kit for specific steps.

### Confocal Microscopy of Lipid Droplets

Cells were inoculated into a 12-well culture plate with about 5,000 cells per well. A 1 mL cell suspension was added to each well. After 24 h of culture, the medium was discarded, and 1 mL of serum-free and double antibody-free medium was added for 12 h. Cells were stimulated with 1.2 m*M* fatty acid for 12 h. Subsequently, cells were cleaned twice with phosphate-buffered saline (**PBS**) and fixed for 30 min with 4% paraformaldehyde. After cleaning with PBS for three times, BODIPY 493/503 dye was added for 30 min in the dark. Cells were washed twice with PBS, the nuclei were then stained with Hoechst (Beyotime Biotechnology, Shanghai, China) for 8 min prior to fluorescent microscopy.

### Reactive Oxygen Species (ROS) Determination

Dilute DCFH-DA in DMEM medium to a final concentration of 10 μm. After treating the cells, wash them twice with PBS, collect the cells in a 15 ml centrifuge tube by trypsinization. Mitochondrial ROS was assayed by labeling cells with 10 m*M* dihydrorhodamine 123 (DHR123, Molecular Probes) for 15 min. Samples were assayed by FACS using a Canto IITM cell sorter (BD Biosciences, New Jersey, USA). The results were analyzed using Cell Quest software (BD Biosciences, New Jersey, USA).

### Statistical Analysis

The experiments were conducted in 4 separate cell preparations from four calves using three replicates per treatment. Data were analyzed using SPSS 22.0 (SPSS Inc., Chicago, IL) and GraphPad Prism 7.00 (GraphPadSoftware, San Diego, CA). Results are reported as means ± standard error of the mean (SEM). Statistical significance was determined using two-tailed unpaired Student's *t*-test or one-way ANOVA followed by Tukey's *post-test* for comparisons between 2 or among more than 2 groups, respectively. Differences were considered significant at *P* < 0.05.

## Results

### Effects of Different Fatty Acids on Oxidative Stress and ER Stress in Primary Calf Hepatocytes

**Figure 1:**
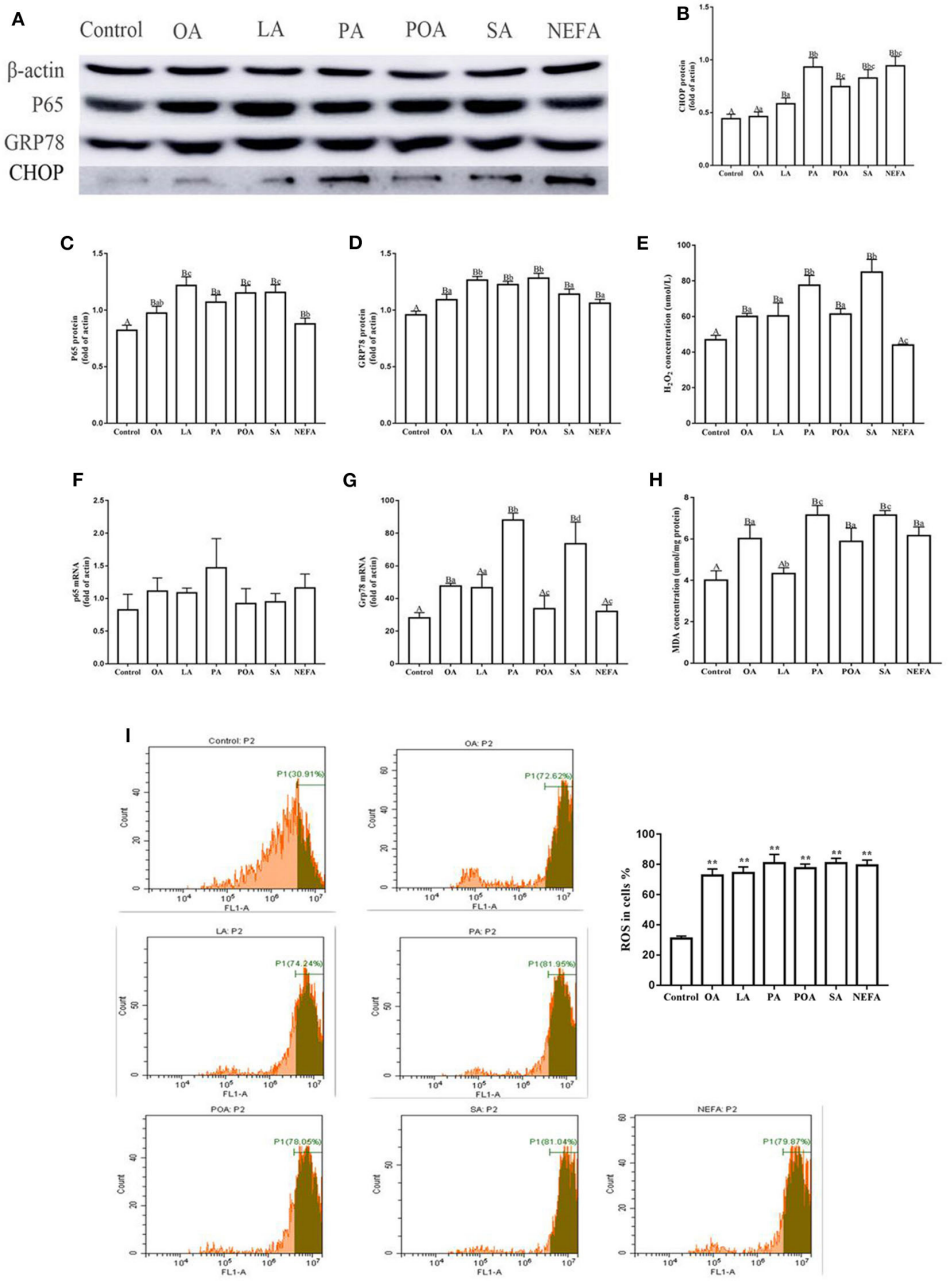
Effects of different fatty acids on stress and inflammatory response of primary hepatocytes. Cells were treated with 1.2 m*M* fatty acids for 12 h (*n* = 3 ell preparations). **(A)** β-actin, P65, GRP78, and CHOP protein expression were assayed by western blotting. Gray-shaded bars represent the amount (%) of β-actin, P65, GRP78, and CHOP in primary hepatocytes. **(B–D)** CHOP, P65, and GRP78 protein expression in primary hepatocytes were assayed by western blotting and reported relative to β-actin. **(F,G)** P65 and GRP78 mRNA expression were assayed by real-time RT-qPCR. **(I)** ROS were assayed by fluorescence microscopy and flow cytometry. Fluorescence intensity is proportional to ROS activity in primary hepatocytes. ^**^p < 0.01 solvent and control group. **(E,H)** H_2_O_2_ (μmol/L) and MDA (μmol/mg protein) were assayed using commercial kits. Four assays were independently performed in each group. Uppercase letter vs. control *p* ≤ 0.05, lowercase letter vs. fatty acid group *p* ≤ 0.05, the same letter vs. no letter mark *p* ≥ 0.05.

The protein abundance of NF-κB (p65) in fatty acid-treated cells increased significantly (Figure 1A; *P* ≤ 0.05), suggesting an inflammatory response. The GRP78 and CHOP protein abundance in the fatty acid-treated groups was greater than those in the control group. CHOP protein abundance in the PA, SA and NEFA groups was greater than those of other groups, and GRP78 mRNA abundance in the PA and SA groups was greater than those of other groups (Figures 1A–D,F,G; *P* ≤ 0.05).

Fatty acid challenge promoted generation of oxidative stress. The content of H_2_O_2_ in the single fatty acid-treated groups was greater than that of the control group, and the content in the PA and SA group was greater compared with other groups (Figure 1E). The MDA content in OA, PA, POA, SA, and NEFA groups was greater than the control and LA groups (Figure 1H). The ROS content in the fatty acid-treated groups was greater compared with the control group (Figure 1I).

### Effects of Fatty Acids on TG Synthesis

**Figure 2:**
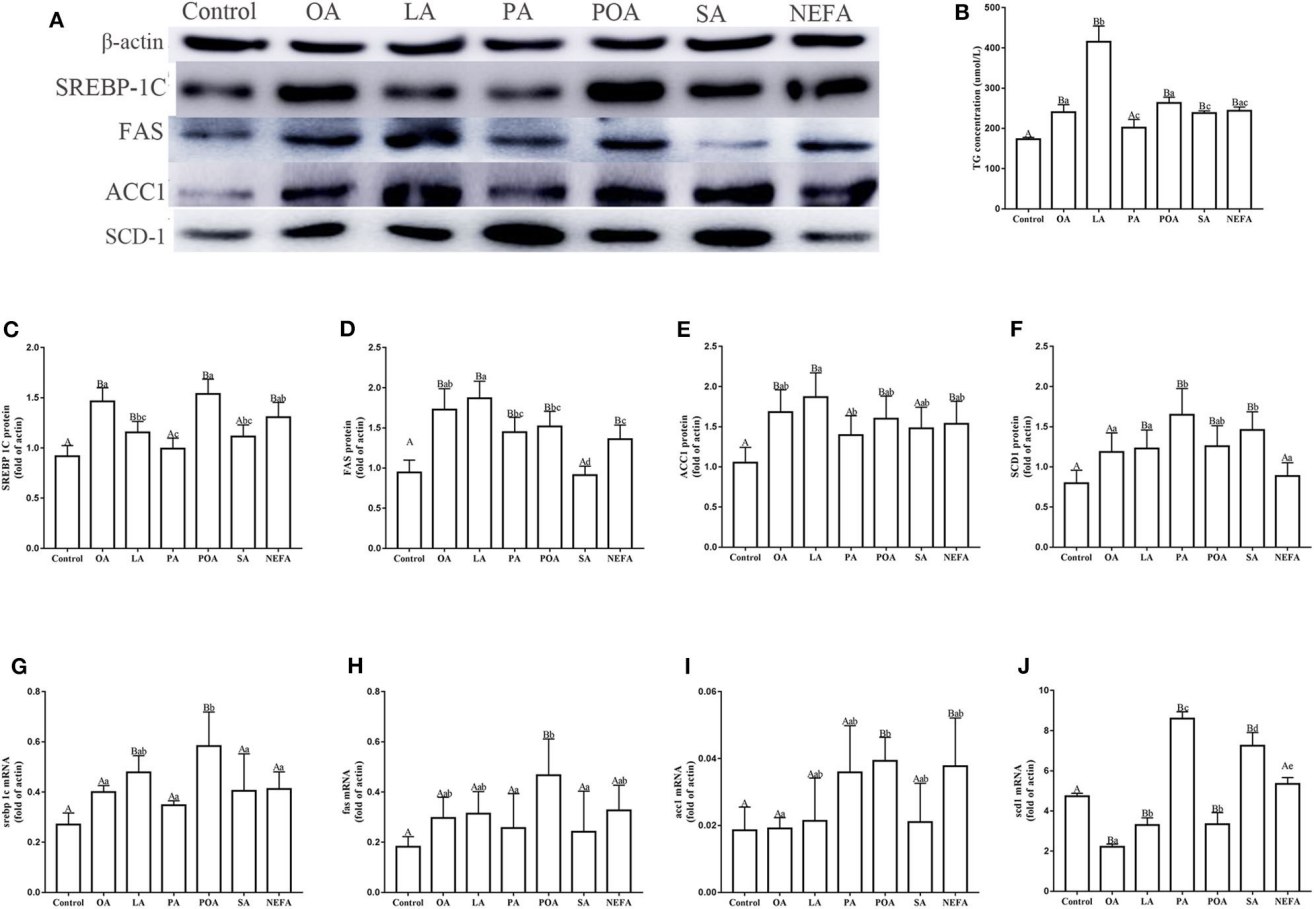
Effects of fatty acids on fat synthesis. Cells were treated with 1.2 m*M* fatty acids for 12 h (*n* = 3 ell preparations). **(A)** β-actin, SREBP-1c, FAS, ACC1, and SCD1 protein expression were assayed by western blotting. Gray-shaded bars represent the amount (%) of β-actin, SREBP-1c, FAS, ACC1, and SCD1 in primary hepatocytes. **(B)** TG concentration was determined by ELISA. **(C–F)** SREBP-1c, FAS, ACC1, and SCD1 protein expression in primary hepatocytes were assayed by western blotting and reported relative to β-actin. **(G–J)** SREBP-1c, FAS, ACC1 and SCD1 mRNA expression were assayed by real-time RT-qPCR. Data are means ± SEM of individual assays. Each assay was performed in triplicate. Uppercase letter vs. control *p* ≤ 0.05, lowercase letter vs. fatty acid group *p* ≤ 0.05, the same letter vs. no letter mark *p* > 0.05.

Protein abundance of SREBP-1c in OA, LA, POA and NEFA groups was greater than the control group; the protein abundance of SREBP-1c in OA group and POA group was significantly higher than that in NEFA group (Figures 2A,C; *P* < 0.05). The mRNA abundance of SREBP-1c in the LA and POA groups was greater compared with other groups (Figure 2G; *P* ≤ 0.05). Compared with the control, protein abundance of FAS with OA, LA, PA, POA, and NEFA groups was greater overall. In addition, FAS protein abundance with OA and LA was greater compared with the NEFA group; abundance of FAS mRNA with POA was greater compared with other groups (Figures 2D,H; *P* ≤ 0.05). Protein abundance of ACC1 with OA, LA, POA, and NEFA was greater compared with the control group. Abundance of ACC1 mRNA with POA and NEFA was greater compared with other groups (Figures 2E,I, *P* ≤ 0.05).

The protein abundance of SCD1 with LA, PA, POA, and SA was greater compared with the control group. In addition, protein and mRNA abundance of SCD1 with PA and SA was greater compared with the NEFA group (*P* ≤ 0.05). Abundance of SCD1 mRNA with OA, LA, and POA was lower compared with both the PA and SA groups (Figures 2F,J). Content of TG with OA, LA, POA, SA, and NEFA groups was greater compared with the control group. Further, TG content with LA was greater compared with other fatty acid groups (Figure 2B).

### Effects of Different Fatty Acids on Lipid Deposition of Primary Calf Hepatocytes

**Figure 3:**
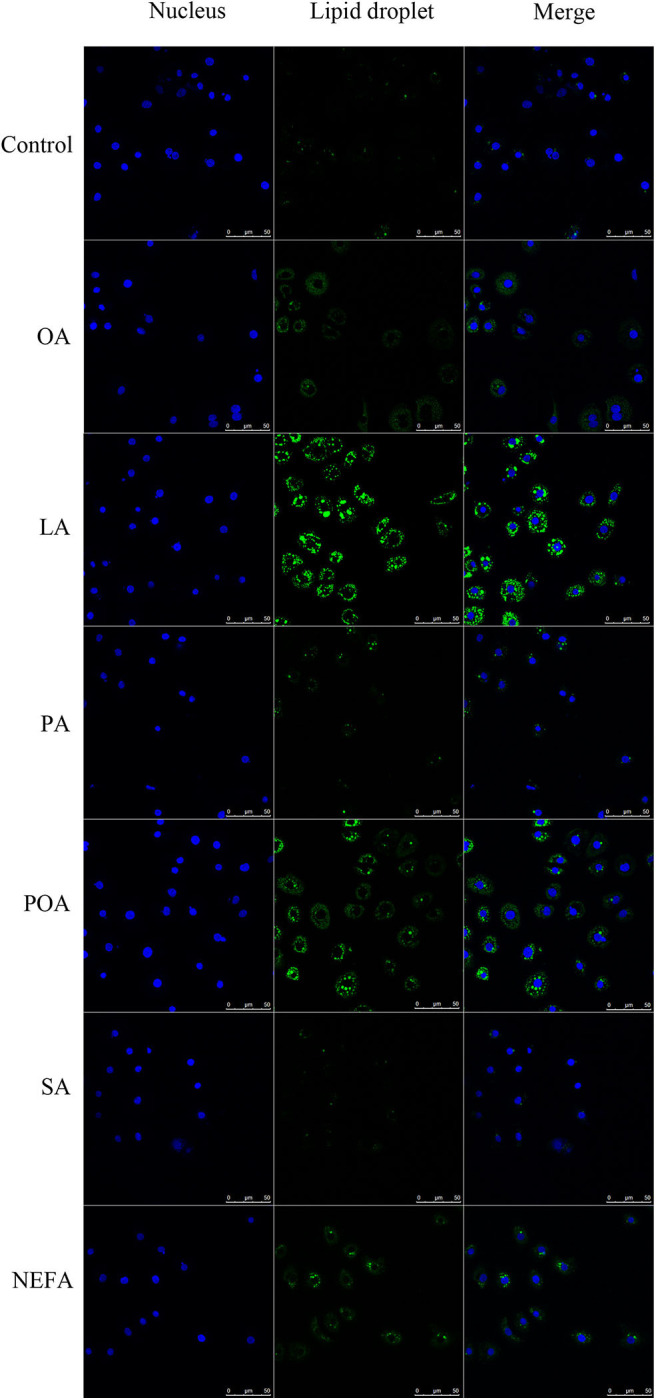
Effects of fatty acids on the formation of lipid droplets. Cells were treated with 1.2 mM fatty acids s for 12 h, then lipid droplets in cells were stained by BODIPY, and cell nuclei were stained by Hoechst. Blue fluorescence indicates cell nuclei; green fluorescence indicates lipid droplets, the intensity of green fluorescence increases with the number of lipid droplets in the cell.

The amount of lipid droplets produced in response to NEFA or individual fatty acids was greater compared with the control group. Further, lipid droplets produced with LA were greater compared with other groups. The amount of lipid droplets produced with OA, LA, POA, and NEFA was greater compared with other groups (Figure 3).

### Assembly and Synthesis of VLDL

**Figure 4:**
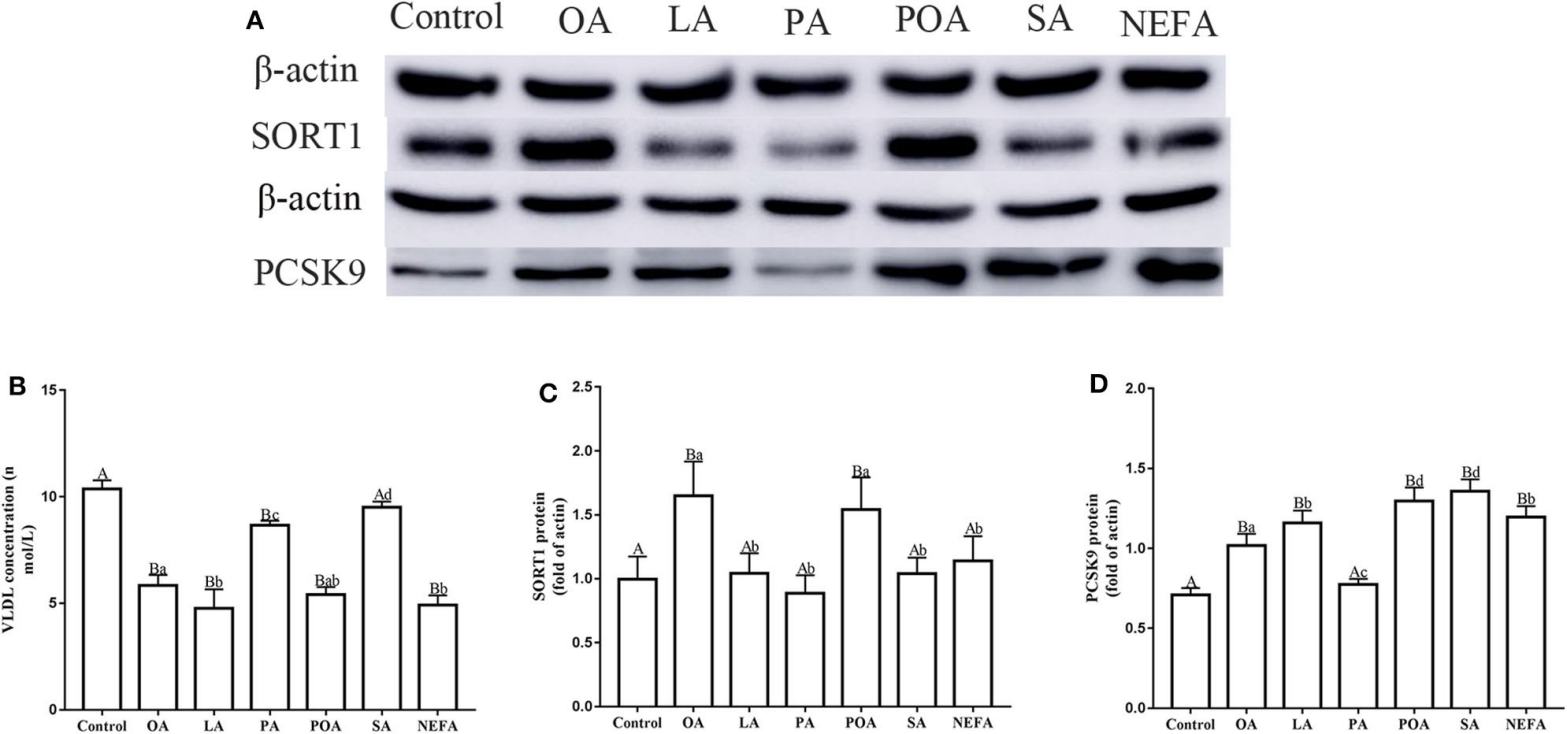
Effects of different fatty acids on the assembly and synthesis of VLDL in primary hepatocytes. Cells were treated with 1.2 m*M* fatty acids for 12 h (*n* = 3 ell preparations). **(A)** β-actin, SORT1, and PCSK9 protein expression were assayed by western blotting. Gray-shaded bars represent the amount (%) of β-actin, SORT1, and PCSK9 protein in primary hepatocytes. **(B)** VLDL concentration was determined by ELISA. **(C,D)** SORT1 and PCSK9 expression were reported relative to that of β-actin. Data are means ± SEM. Each assay was performed in triplicate. Uppercase letter vs. control *p* ≤ 0.05, lowercase letter vs. fatty acid group *p* ≤ 0.05, and same letter vs. no letter mark *p* ≥ 0.05.

**Figure 5:**
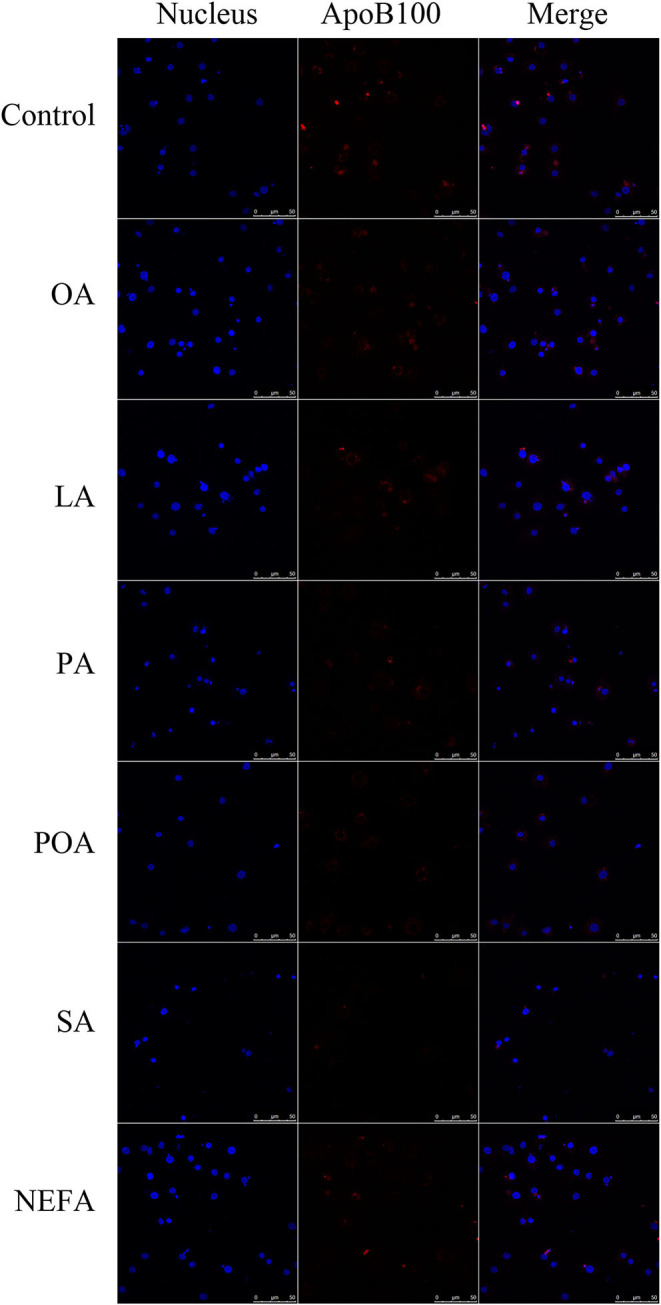
The effect of fatty acids on the expression of ApoB100 in primary hepatocytes. Cells were treated with 1.2 m*M* fatty acids for 12 h. The ApoB100 in cells were stained by BODIPY, and cell nuclei were stained by Hoechst. Blue fluorescence indicates cell nuclei; red fluorescence indicates ApoB100, the intensity of red fluorescence increases with the expression of ApoB100 in the cell.

Except for SA, concentration of VLDL in the fatty acid-challenged groups was lower compared with the control group (Figure 4B). Protein abundance of SORT1 with OA and POA was greater compared with other groups (Figures 4A,C). Protein abundance of PCSK9 with OA, LA, POA, SA, and NEFA was greater compared with the control group (Figures 4A,D). Fluorescence intensity of ApoB100 with fatty acid addition group was lower compared with the control group, with the response in the PA and SA groups being lower than other fatty acids (Figure 5).

## Discussion

In dairy cows, fatty liver is characterized by high plasma NEFA concentrations and occurs most frequently during periods of NEB. High influx of NEFA into liver could promote oxidative stress, altered lipid metabolism (26), and hepatic steatosis (27). *In vitro* studies with HepG2, HuH7, and WRL68 hepatocytes indicated that elevated supply of exogenous oleic acid and palmitic acid caused hepatic steatosis and hepatocyte damage (24). However, a correlation between specific plasma fatty acids and liver damage during early lactation in dairy cows is unclear. Therefore, in our study, the characteristics of the most common NEFA in blood plasma (POA, SA, OA, and LA) induced lipid deposition and injury in primary calf hepatocytes was analyzed.

Overall, the SFA used in the present study led to greater abundance of the ER stress markers GRP78 and CHOP, oxidative stress markers H_2_O_2_ and MDA, and the inflammation transcription factor NF-κB subunit p65 suggesting the induction of severe hepatocyte damage. Endoplasmic reticulum stress is caused by accumulation of misfolded proteins (28). In the process of protein folding, ROS are produced as by-products, which leads to alterations in redox balance and oxidative stress (29). NF-κB is a transcription factor that regulates gene expression and can affect cell survival, growth, proliferation, and apoptosis. Its signaling plays a key role in NAFLD (30). Persistent oxidative stress and ER stress activates apoptotic pathways and triggers hepatocyte damage (31). *In vivo*, the existence of ER stress in the liver of severe fatty liver cows may presage its participation in fatty liver progression in dairy cows (4).

Mitochondrial activities, including beta-oxidation of free fatty acids, production of ATP, and ROS are all central to homeostatic regulation of lipid and energy metabolism in hepatocytes (32). In addition, compounds such as ROS are important for the regulation of apoptosis (33). *In vitro*, compared with OA, exogenous PA led to greater ROS accumulation in rat hepatoma cells, a response partly caused by the inability of mitochondria to handle large amounts of exogenous long-chain fatty acids, which often leads to mitochondrial dysfunction (34, 35). The content of MDA produced by lipid peroxidation by ROS is also a marker for oxidative stress, thus, the greater concentration of MDA in response to exogenous SFA in the present study underscored the existence of serious oxidative stress. *In vivo* study, mid-lactating cows was supplemented SPA, α-linolenic acid, and conjugated linoleic acid for 6 weeks, respectively, it was found that SFA supplemented cows showed more serious oxidative stress with a lower plasma glutathione peroxidase activity than in α-linolenic acid, and lower plasma concentration of β-carotene than in α-linolenic acid and conjugated linoleic acid (36). Our previous *in vitro* studies confirmed that increases in ROS during mitochondrial metabolism can further enhance ER stress (37).

The greater content of lipid droplets and triacylglycerol in response to UFA compared with SFA along with the greater abundance of SREBP-1c, FAS, and ACC1 suggested a potential protective role by channeling utilization of fatty acids for TG synthesis to help reduce lipid toxicity (38). These responses agree with previous data indicating that exogenous USFA can activate SREBP-1c transcription and upregulate FAS and ACC1 (39). From a mechanistic standpoint, this effect of exogenous UFA might involve loss of torsinA activity, which was shown to control budding of lipid droplets from the ER and, thus, controlling availability of fatty acids to mitochondria and the rates of fatty acid oxidation (40). Future studies exploring the role of lipid droplet-associated proteins in the control of mitochondrial fatty acid metabolism appear warranted (39).

The VLDL is essential for export of endogenous TG out of the liver, and the lower concentration of VLDL observed primarily with USFA and NEFA suggested a potentially negative impact on the export process. This idea is supported by the greater abundance of ApoB100 and SORT1 coupled with greater concentration of TG. ApoB100 is essential for VLDL assembly, aiding in the “lipidation” of nascent VLDL through a concerted mechanism with microsomal triglyceride transfer protein (**MTP**) in the lumen of the ER. SORT1 is an intracellular sorting receptor for ApoB100. It interacts with ApoB100 in the Golgi and facilitates the formation and hepatic export of ApoB100-containing lipoproteins (41). From the present data, it is possible that the elevated concentration of exogenous fatty acids overwhelmed the VLDL synthesis machinery, which agrees with a previous study demonstrating changes in ApoB100 secretion as a function of fatty acid availability (42). In that context, we speculate that the greater abundance of PCSK9 in response to exogenous USFA and SFA (except SA) was a response to counteract the impairment in VLDL export. In mice, PCSK9 interacts directly with cytoplasmic ApoB100 and prevents its degradation via the autophagosome/lysosome pathway (43). Thus, it is possible that abundance of PCSK9 is another component of the mechanisms that help control liver fat accumulation. However, *in vivo* study found that feeding LA and linolenic acids as unprotected oilseeds increased dry matter intake over time at a greater extent for cows fed PA, improved the energy status, and lowered hepatic lipids and triacylglycerol contents, which may contribute to enhance the health status of transition dairy cows (44).

**Figure 6:**
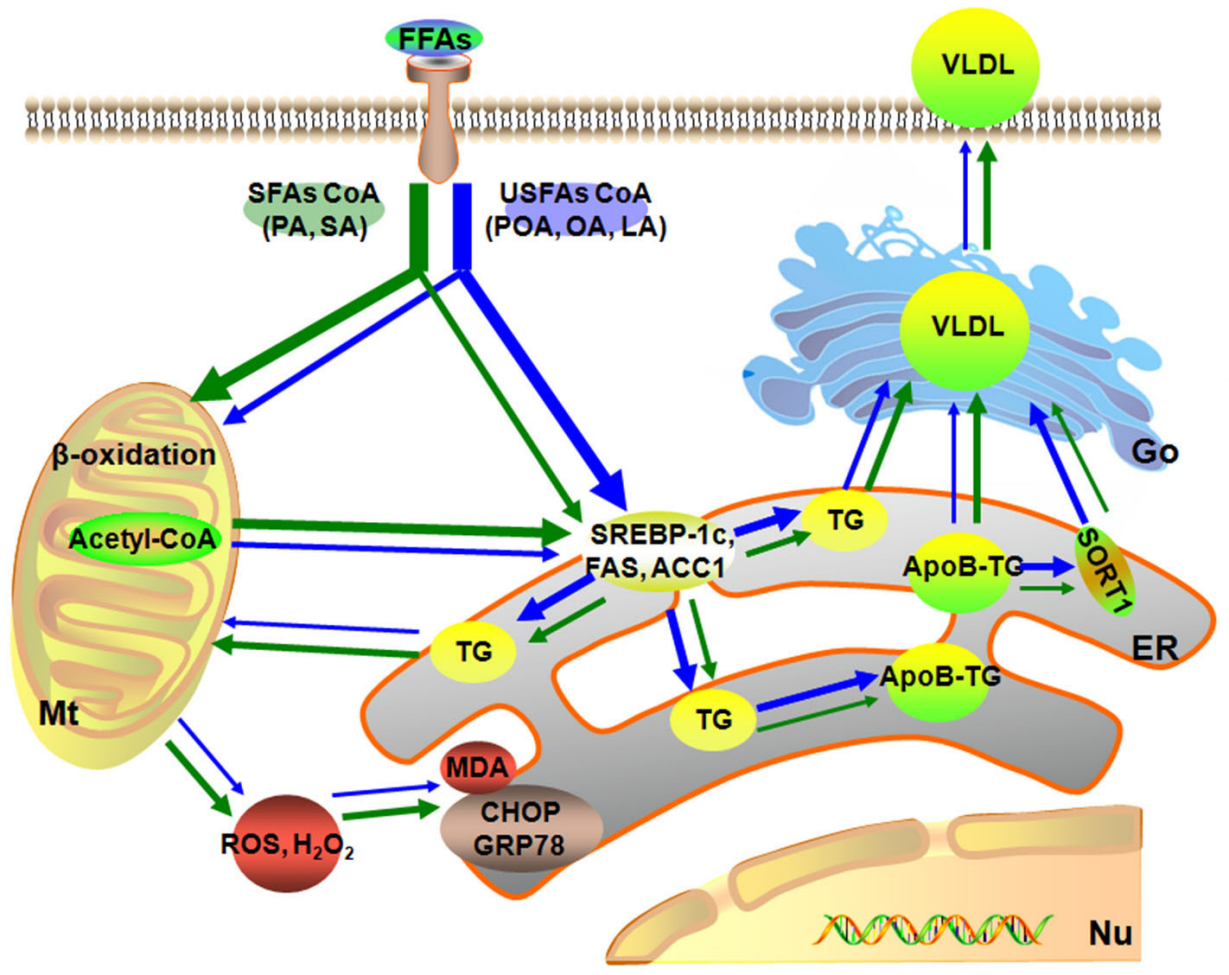
Model of different fatty acids induced lipid accumulation and liver injury characteristics of primary calf hepatocytes. The green arrow represents the SFAs metabolic, blue arrow represents the USFAs metabolic. The thickness of the arrow represents the level of fatty acid metabolism. Mt, mitochondria; Nu, nucleus; ER, endoplasmic reticulum.

In summary, a dose of 1.2 mmol/L of individual or mixture of fatty acids induced different degrees of lipid accumulation and hepatocyte damage. Compared with USFA (POA, OA, and LA), exogenous SFA (PA and SA) led to more pronounced oxidative and ER stress. In contrast, exogenous USFA led to greater TG accumulation driven in part by an induction of lipogenesis along with ApoB100 and SORT1. That effect, however, was associated with lower VLDL discharge into cell culture medium (Figure 6). Overall, the data suggest that type of exogenous long-chain fatty acid can induce distinct effects at the level of the hepatocyte. Although many published studies using hepatocytes from calves to address metabolism of the adult ruminant liver (45–47), whether these responses could be induced *in vivo* by feeding specific fatty acids remained to be determined.

## Data Availability Statement

All datasets generated for this study are included in the article/supplementary material.

## Ethics Statement

The animal study was reviewed and approved by the Ethics Committee for the Use and Care of Animals, Heilongjiang Bayi Agricultural University.

## Author Contributions

BZ, WY, and CXu designed this project. RL, SW, ZD, YZ, and XM performed the experiments and analyzed the results. JL and CXi revised the manuscript. BZ and WY drafted this manuscript. All authors revised final version of the manuscript.

## Conflict of Interest

The authors declare that the research was conducted in the absence of any commercial or financial relationships that could be construed as a potential conflict of interest.

## Abbreviations

ACC1: acetyl coenzyme A carboxylase 1
ApoB100: Apolipoprotein B100
CHOP: C/EBP homologous protein
ER: endoplasmic reticulum
FAS: fatty acid synthase
FBS: fetal calf serum
GRP78: glucose-regulated protein
LA: linoleic
LCFA: long-chain fatty acids
LDL-C: low density lipoprotein-cholesterol
MDA: malonaldehyde
MTP: microsomal triglyceride transfer protein
NASH: non-alcoholic steatohepatitis
NEFA: non-esterified fatty acid
OA: oleic
PA: palmitic
PBS: phosphate-buffered saline
PCSK9: proprotein convertase subtilisin/kexin type 9
POA: palmitoleic
PVDF: polyvinylidene difluoride
ROS: reactive oxygen species
SA: stearic
SREBP-1c: Sterol regulatory element-binding protein-1c
SORT1: Sortilin
TG: triacylglycerol
UPR: unfolded protein reaction
VLDL: lower very low density lipoprotein.
